# Diagnostic accuracy of heart rate variability as a screening tool for mild neurocognitive disorder

**DOI:** 10.3389/fnagi.2024.1498687

**Published:** 2024-12-17

**Authors:** Julia Czopek-Rowinska, Eling D. de Bruin, Patrick Manser

**Affiliations:** ^1^Motor Control and Learning Group, Institute of Human Movement Sciences and Sport, Department of Health Sciences and Technology, ETH Zurich, Zurich, Switzerland; ^2^Department of Health, OST - Eastern Swiss University of Applied Sciences, St. Gallen, Switzerland; ^3^Division of Physiotherapy, Department of Neurobiology, Care Sciences and Society, Karolinska Institutet, Stockholm, Sweden

**Keywords:** area under curve, cognitive dysfunction, dementia, depression, executive function, ROC curve, secondary prevention, sensitivity and specificity

## Abstract

**Background:**

Mild neurocognitive disorder (mNCD) is recognized as an early stage of dementia and is gaining attention as a significant healthcare problem due to current demographic changes and increasing numbers of patients. Timely detection of mNCD provides an opportunity for early interventions that can potentially slow down or prevent cognitive decline. Heart rate variability (HRV) may be a promising measure, as it has been shown to be sensitive to cognitive impairment. However, there is currently no evidence regarding the diagnostic accuracy of HRV measurements in the context of the mNCD population. This study aimed to evaluate the diagnostic accuracy of vagally-mediated HRV (vm-HRV) as a screening tool for mNCD and to investigate the relationship between vm-HRV with executive functioning and depression in older adults who have mNCD.

**Methods:**

We retrospectively analyzed data from healthy older adults (HOA) and individuals with a clinical diagnosis of mNCD with a biomarker-supported characterization of the etiology of mNCD. Diagnostic accuracy was evaluated using receiver operating characteristic curve analysis based on the area under the curve. Sensitivity and specificity were calculated based on the optimal threshold provided by Youden’s Index. Multiple linear regression analyses were conducted to investigate the relationship between vm-HRV and executive functioning and depression.

**Results:**

This analysis included 42 HOA and 29 individuals with mNCD. The relative power of high frequency was found to be increased in individuals with mNCD. The greatest AUC calculated was 0.68 (with 95% CI: 0.56, 0.81) for the relative power of high frequency. AUCs for other vm-HRV parameters were between 0.53 and 0.61. No consistent correlations were found between vm-HRV and executive functioning or depression.

**Conclusion:**

It appears that vm-HRV parameters alone are insufficient to reliably distinguish between HOA and older adults with mNCD. Additionally, the relationship between vm-HRV and executive functioning remains unclear and requires further investigation. Prospective studies that encompass a broad range of neurocognitive disorders, HRV measurements, neuroimaging, and multimodal approaches that consider a variety of functional domains affected in mNCD are warranted to further investigate the potential of vm-HRV as part of a multimodal screening tool for mNCD. These multimodal measures have the potential to improve the early detection of mNCD in the future.

## 1 Introduction

### 1.1 Background

Mild neurocognitive disorder (mNCD; American Psychiatric Association, 2013; World Health Organization, 2018), also known as mild cognitive impairment (MCI; Bermejo-Pareja et al., 2020), is a precursor to dementia (Petersen et al., 2018) that is gaining attention as a significant healthcare and societal problem due to current demographic changes with increasing number of patients (Fendrich and Hoffmann, 2007) and healthcare costs (Prince et al., 2015). More than 60% of people with dementia are not detected worldwide (Lang et al., 2017) and there are currently no curative treatments for neurocognitive disorders (Demurtas et al., 2020). However, timely detection of mNCD provides an opportunity for early interventions, such as lifestyle modifications (Livingston et al., 2020) and therapies (i.e., exercise interventions; Huang et al., 2022), that can potentially slow down or prevent cognitive decline (Sabbagh et al., 2020; Veronese et al., 2023; Manser et al., 2024), contributing toward active solutions to this healthcare problem. Hence, in the present day, researchers and healthcare practitioners’ primary focus should be the early detection of mNCD (Langa and Levine, 2014; Shah et al., 2016).

Several research approaches have been undertaken to diagnose mNCD, including magnetic resonance imaging, positron emission tomography (Mosconi, 2005; Talwar et al., 2021), cerebrospinal fluid biomarkers (Papaliagkas et al., 2023), and neurocognitive assessments (American Psychiatric Association, 2013). It is recommended that these procedures be conducted only when there is a strong indication of mNCD, as they can be invasive and costly. Such a clinical indication can be effectively provided by a screening tool with high diagnostic accuracy. The most frequently used screening tools for individuals with suspected mNCD include cognitive screening instruments like the Mini-Mental State Examination (MMSE; Karimi et al., 2022) and the Montreal Cognitive Assessment (MoCA; Nasreddine et al., 2005), but there are many alternatives such as the Memory Alteration Test and the Quick Mild Cognitive Impairment (Qmci) test (Breton et al., 2019). Most of them exhibit high sensitivity for mNCD (Nasreddine et al., 2005) and can be administered by a clinician over a short period (Breton et al., 2019). However, the major limitation lies in inadequate specificity at their recommended cut-off within these tests (Ozer et al., 2016; Islam et al., 2023), which in turn might lead to potential harms and rising costs that have not yet been adequately studied (Breton et al., 2019). This warrants a screening tool with high diagnostic accuracy which allows accurate discrimination between at-risk and healthy individuals in a quick and easy manner.

Heart rate variability (HRV) has been suggested by several studies as a potential biomarker of cognitive impairment (Forte et al., 2019; Nicolini et al., 2022; Grässler et al., 2023). HRV is a measure of the variation in the time intervals between consecutive heartbeats and is generated by heart-brain interactions that reflect a dynamic interplay between the sympathetic and parasympathetic branches of the autonomic nervous system (Shaffer and Ginsberg, 2017). HRV can be measured with good validity and reliability using wearable sensors (Nunan et al., 2009; Board et al., 2016; Dobbs et al., 2019). It is therefore readily accessible in everyday life, enabling continuous monitoring through wearable devices, and might offer a promising physiological marker to detect mNCD.

According to the “neurovisceral integration” model, cardiac vagal tone, indexed by vagally-mediated heart rate variability (vm-HRV), can indicate the functional integrity of the central autonomic network (CAN) implicated in emotion–cognition interactions (Thayer and Lane, 2000), and is positively correlated with prefrontal cortical performance (Thayer, 2009). Brain areas in CAN, including prefrontal cortical regions, have been shown to be affected in mNCD patients (Van Dam et al., 2013; Xu et al., 2020). Accordingly, recent studies found notably reduced vagally-mediated HRV indices in mNCD individuals compared to healthy older adults (HOA; da Silva et al., 2018; Cheng et al., 2022; Grässler et al., 2023), suggesting the presence of autonomic dysfunction in mNCD (da Silva et al., 2018; Grässler et al., 2023). While these findings suggest that vm-HRV is sensitive to mNCD, there is currently no evidence regarding the diagnostic accuracy of HRV measurements in the context of the mNCD population.

### 1.2 Objectives

The primary objective of this study was to evaluate the diagnostic accuracy of vm-HRV as a screening tool for mNCD. As a secondary objective, the relationship between vm-HRV with executive functioning and depression was investigated in older adults diagnosed with mNCD to further explore emotion-cognition interactions with vm-HRV in mNCD (Thayer and Lane, 2000; Ha et al., 2015).

## 2 Materials and methods

### 2.1 Study design and setting

A retrospective analysis was conducted on the baseline data from three studies. These studies include a cross-sectional study that evaluated the test–retest reliability and validity of phasic vm-HRV responses during exergaming in HOA (Manser and de Bruin, 2024a) and two intervention studies that assessed the feasibility (Manser et al., 2023b) and effectiveness (Manser et al., 2023a; Manser and de Bruin, 2024b) of a novel technology-enhanced training concept for the secondary prevention of mNCD. This retrospective analysis study is reported according to STARD 2015 guidelines (checklist see Supplementary material; Cohen et al., 2016).

In the cross-sectional study, a consecutive sample of HOA was recruited from January 2021 to June 2021 through collaborations with healthcare institutions in the wider Zurich area by handing out leaflets to interested individuals. All interested individuals were fully informed about the study by trained investigators from our research team by providing verbal explanations and an information sheet before providing written informed consent. In the two intervention studies, older adults with mNCD were recruited from July 2021 to October 2023 in collaboration with (memory) clinics in Cantons Zurich and St. Gallen, Switzerland. Suitable patients were either identified from medical records and patient registries of (memory) clinics or from recent clinical diagnostics performed by their medical doctors or therapists authorized to search medical records.

To ensure diversity, equity, and inclusion, all patients referred to us by the clinical recruitment partners as well as all interested HOAs were fully considered for participation in the respective studies. All data was collected from January 2021 to November 2023. After completing recruitment and obtaining written informed consent, participants underwent eligibility screening (see Table 1) and appointments for measurements for eligible individuals were scheduled. During the first scheduled appointment, baseline assessments were conducted.

**Table 1:** Description of all eligibility criteria.

Inclusion criteria	Exclusion criteria
Participants fulfilling the following inclusion criteria were eligible:	The presence of any of the following criteria led to exclusion:
clinical diagnosis of ‘mild neurocognitive disorder’ according to International Classification of Diseases 11th Revision (ICD-XI; World Health Organization, 2018) or the latest Diagnostic and Statistical Manual of Mental Disorders 5th Edition (DSM-5®; American Psychiatric Association, 2013) or healthy (based on self-report) older adults (≥ 60 years) German speaking able to stand for at least 10 min without assistance	Mobility impairments (i.e., gait and balance) that prevent experiment participation Presence of additional, clinically relevant (i.e., acute or symptomatic or both) neurological disorders (i.e., epilepsy, stroke, multiple sclerosis, Parkinson’s disease, brain tumors, or traumatic disorders of the nervous system) Presence of any other unstable or uncontrolled diseases (e.g., uncontrolled high blood pressure and progressing or terminal cancer)

### 2.2 Eligibility criteria

HOA were eligible if they were ≥ 60 years old and healthy according to self-report. Older adults with mNCD were eligible if they were clinically diagnosed with mNCD according to the Diagnostic and Statistical Manual of Mental Disorders 5th Edition (World Health Organization, 2018) or International Classification of Diseases 11th Revision (American Psychiatric Association, 2013) as well as having a biomarker-supported characterization of the etiology of mNCD. Besides these inclusion criteria for the characterization of the population (HOA and mNCD), we used identical eligibility criteria for all three studies, as shown in Table 1.

### 2.3 Sample size

The sample size was determined by the available datasets from studies conducted under the “Brain-IT” project, and therefore, no prior sample size calculation was performed. The available data comprised 80 participants, including 43 HOA and 37 older adults with mNCD.

### 2.4 Outcomes

#### 2.4.1 Primary outcome: vagally mediated HRV

Resting vm-HRV was measured following the same protocol in all three studies. The measurement took place at the beginning of the session, in a quiet room at room temperature. Participants were instructed to sit comfortably on a chair with feet flat on the floor, knees at a 90° angle, hands resting on their thighs (palms facing upward), eyes closed, and refraining from speaking (Laborde et al., 2017). The acclimatization period lasted 5 min, followed by a 5-min resting measurement - the recommended duration for short-term recordings - during which the start of the resting measurement was not announced to the participant (Malik, 1996; Laborde et al., 2017). For measuring resting vagal tone, a heart rate monitor (Polar M430) and sensor (Polar H10) were employed, a valid tool for HRV assessment (Giles et al., 2016). The data obtained were analyzed using the validated Kubios HRV Premium Software (Kubios Oy, Kuopio, Finland, Version 3.4; Tarvainen et al., 2014) following the procedures defined in the study protocol by Manser et al. (2023a).

More specifically, a sampling rate of 1,000 Hz was used to provide a temporal resolution of 1 ms for each R-R interval (Williams et al., 2017). R-R data was transmitted to Kubios HRV Premium (Kubios Oy, Kuopio, Finland, Version 3.4) for analysis. Kubios HRV is a scientifically validated software for HRV analysis and has achieved a gold-standard status in scientific research (Tarvainen et al., 2002; Niskanen et al., 2004; Tarvainen et al., 2014; Lipponen and Tarvainen, 2019). The automatic beat correction algorithm and noise handling provided by the software was used to correct for artifact and/or ectopic beats. The algorithm was validated for resting measurements (Lipponen and Tarvainen, 2019). The entire 5-min resting measurement was analyzed. After removing inter-beat-interval time series non-stationarities by detrending analysis using the smoothness priors method approach (settings: detrending method = smoothn priors, Lambda = 500, fc = 0.035 Hz), mean values of mainly vm-HRV indices were calculated for each segment. We analyzed several vm-HRV indices, including the mean R-R time interval (mRR), root mean square of successive RR interval differences (RMSSD), the absolute power of the high-frequency (0.15–0.4 Hz; HF) band, the relative power of HF (in normal units; HF in n.u. = HF in ms^2^)/ [total power in ms^2^–very low frequency (0.00–0.04 Hz in ms^2^)], and the Poincaré plot standard deviation perpendicular to the line of identity (SD1; Ernst, 2017; Laborde et al., 2017; Shaffer and Ginsberg, 2017; Tarvainen et al., 2018). Additionally, the parasympathetic nervous system tone index (PNS-Index) was calculated that compares PNS activity to normal resting values (Tarvainen et al., 2018).

To reduce the impact of confounding factors on HRV, participants were instructed to follow a normal sleep routine, avoid intense physical activities and alcohol consumption 1 day before the experiment, and refrain from coffee as well as food consumption at least 2 h before the measurements (Laborde et al., 2017). Two qualified operatives, trained in proper application of measurement tools and protocols, assisted with each measurement.

#### 2.4.2 Secondary outcomes: executive functions and depression

We used data from standardized neuropsychological assessments that evaluated executive functions. For an overview, we used data from the HOTAP picture-sorting test (HOTAP) that assessed planning ability (Menzel-Begemann, 2009), a computerized version of the Trail Making Test-Part B (PEBL-TMT-B) that evaluated cognitive flexibility (Mueller, 2014; Mueller and Piper, 2014), and a computerized version of the Digit Span Backward test PEBL-DSB that assessed working memory (Croschere et al., 2012; Mueller, 2014; Mueller and Piper, 2014).

Depression was evaluated using the short version of the Depression, Anxiety, and Stress Scale-21 (DASS-21; Lovibond and Lovibond, 1995; Lovibond, 1995; Henry and Crawford, 2005) with a subscale depression cutoff score of ≥ 5 indicating clinically relevant depression (Nilges and Essau, 2015).

All assessments were administered and evaluated in accordance with published guidelines or detailed working instructions. For further information on these assessments and measurement procedures, please refer to the study protocol of our RCT (Manser et al., 2023a).

#### 2.4.3 Baseline factors

Baseline factors were collected through demographic data including sex, age, body mass index (BMI), years of education, physical activity [i.e., time spent in at least moderate level of physical activity per week or metabolic equivalent of task (MET)-minutes per week], measured with the international physical activity questionnaire (IPAQ)(Forde, 2018; Craig et al., 2003), medication intake (yes/no), etiology of mNCD (mainly mNCD due to Alzheimer’s Disease, mild vascular NCD, mild Frontotemporal NCD or mNCD with Lewy Bodies) and global cognitive performance, measured with the validated German version (Manser and De Bruin, 2024c) of the Quick Mild Cognitive Impairment Screen total score (Qmci).

### 2.5 Statistics

Statistical analysis was performed using RStudio (Version 2023.06.1 + 524). Descriptive statistics were conducted on all outcome variables. Data was reported as mean ± standard deviation for data fulfilling all the assumptions that would subsequently justify parametric statistical analyses, and median (interquartile range) for data not fulfilling all these assumptions. The Shapiro–Wilk test was used to check the normality of data (significance level *p* ≤ 0.05, two-tailed). Between-group differences were tested using a *t*-test (parametric analyses) or the Mann–Whitney *U* test (non-parametric analyses), and the Chi-squared test for categorical data. Effect sizes were evaluated using Cohen’s D, Wilcoxon R, and phi, respectively. For the analysis of HRV data, we excluded invalid measurements from analysis that were a result from either missing data resulting from low quality data (≥ 5% of beats corrected by the automatic beat correction algorithm of Kubios HRV Premium), measurement failure (Polar Sensor failure), or any HRV values identified as extreme by the R function “*identify_outliers* “.

#### 2.5.1 Primary outcome: diagnostic accuracy of vm-HRV

To answer the primary research question, the area under the curve (AUC) from receiver operating characteristic (ROC) curve analysis (Carter et al., 2016) with the optimal cut-off score calculated with Youden’s J statistic (Youden index; Youden, 1950) were used to quantify the overall diagnostic accuracy of vm-HRV. These calculations were performed through the *pROC* package available in RStudio for ROC curve generation and the *OptimalCutpoints* package for Youden’s index calculation. The *Optimal.cutpoints* function’s “*direction*” element was adjusted based on the HRV value ratio in the HOA and mNCD groups from the descriptive statistics. The “>“symbol was used when the baseline values in the HOA group were higher, and “<“was used when the values in the mNCD group were higher. The outcome variable of the analyses were sensitivity, specificity, and AUC with 95% confidence intervals (CIs). An AUC value of 0.9–0.99 was interpreted to indicate excellent discriminatory ability, 0.8–0.89 a good discriminatory ability, 0.7–0.79 a fair discriminatory ability, and 0.51–0.69 a poor discriminatory ability. An AUC of 0.7 was set as an acceptable level of diagnostic accuracy (Carter et al., 2016).

#### 2.5.2 Secondary outcome: vm-HRV and executive functions and depression

To answer the secondary research questions, multiple linear regression analyses were conducted (Field et al., 2012). Regression models were designed with the outcome variables executive functioning (i.e., HOTAP-A, PEBL-TMT-B, and PEBL-DSB) or depression (i.e., DASS-21 - score of subscale ‘depression’) and the predictor variable the assessed vm-HRV parameters (i.e., mRR). The *leaps* package and ‘regsubsets’ function were used to conduct all-subsets regression to select the covariates with the smallest AIC (Akaike Information Criterion) from the following variables: age, gender, BMI, years of education, and physical activity (PA; Polyakova et al., 2014; Au et al., 2017; Domènech-Abella et al., 2018; Song et al., 2019; Li et al., 2022; Montemurro et al., 2023). Age was only used as a covariate for executive functioning (Vicini-Chilovi et al., 2010). Based on the given sample size and the rule of thumb that at least 10 participants are needed per predictor variable or covariate (Field et al., 2012), two covariates were chosen for each of the 28 regression models (Harrell, 2001). As PA was assessed with 2 different methods, PA from the Pilot RCT study was recalculated (Manser et al., 2023b) by using 5.5 METs as a moderate intensity activity equivalent for the elderly population (Hall et al., 2013) and multiplied with min/week. The fit of the model was assessed with the coefficient of determination, R^2^.

## 3 Results

### 3.1 Baseline data

Out of the initial 80 participants, 71 were included in the analysis because of either missing (*n* = 5) or low-quality HRV data (*n* = 4). The characteristics of the study population are presented in Table 2, descriptive statistics on vm-HRV are reported in Table 3 and statistics on cognitive and psychological tests are in Table 4. A significant difference between groups was revealed with a medium effect size in terms of age (Wilcoxon *R* = 0.42) and relative power of HF (*R* = 0.31) and large effect size in terms of medication intake (phi = 0.57, *p* < 0.05). There were no adverse events related to any of the study’s measurements.

**Table 2:** Baseline demographic and clinical characteristics of study participants.

	Unit	HOA (*n* = 42)	mNCD (*n* = 29)	Between group difference	
				*p*-value^(1)^	Effect Size^(2)^
Sex	(% female)	59.5% (*n* = 25)	37.9% (*n* = 11)	0.09	0.22
Age	(years)	67.5 (7.0)	76.5 (7.8)	< 0.001*	0.42
Medication	(% yes)	36.0% (*n* = 15)	93.0% (*n* = 27)	< 0.001*	0.57
BMI	kg/m^2^	23.4 ± 3.3	23.6 ± 2.70	0.9	0.03
Physical Activity	MET-min/week	1851 ± 1,161	1961 ± 1,550	0.88	0.04
Education	years	15.9 ± 4.4	15.0 ± 4.14	0.47	0.19
Qmci	total score	79.8 ± 8.3	55.6 ± 11.1	< 0.001*	2.42
mNCD ethology			72% AD (*n* = 21)		
			17% mVD (*n* = 5)		
			7% mFD (*n* = 2)		
			3% LB (=1)		

(1) *p-values for the between-group differences tested with an independent t-test or Mann–Whitney U test in case the data are not normally distributed, or Fisher’s exact test for categorical variables.*

(2) Effect size estimates for the between-group differences tested with an independent *t*-test (effect size Pearson r) or Mann–Whitney U test [effect size Spearman rho (rs)] in case the data are not normally distributed, or Fisher’s exact test for categorical variables (odds ratio).

Data are reported as mean ± standard deviation for data fulfilling all the assumptions that would subsequently justify parametric statistical analyses data and as median (IQR) for data not fulfilling all of these assumptions. BMI, body mass index; AD, Alzheimer’s Disease; mVD, mild vascular neurocognitive disorder; mFD, mild Frontotemporal neurocognitive disorder; LB, Lewy Body. * = statistically significant at *p* < 0.05.

**Table 3:** Baseline vm-HRV and heart rate of participants.

	Unit	HOA (*n* = 42)	mNCD (*n* = 29)	Between group difference	
				*p*-value	Effect size
mRR	ms	849.11 ± 151.30	843.21 ± 135.59	0.62	0.12
RMSSD	ms	9.95 (8.43)	13.70 (14.95)	0.69	0.05
pNN50	%	0.00 (0.29)	0.00 (2.76)	0.11	0.19
HF	ms^2^	36.50 (63.25)	56.00 (135.25)	0.73	0.04
HF	n.u. (normalized unit)	30.74 (25.50)	47.95 ± 23.74	0.009*	0.31
SD1	ms	7.05 (5.95)	9.70 (10.63)	0.69	0.05
PNS	z-score	−1.13 ± 0.90	−1.02 (1.16)	0.43	0.09
Heart rate	bpm	72.82 ± 12.71	72.08 ± 10.58	0.96	0.01

Data are reported as mean ± standard deviation for data fulfilling all the assumptions that would subsequently justify parametric statistical analyses data and as median (IQR) for data not fulfilling all of these assumptions. mRR, mean R-R time interval; RMSSD, root mean square of successive RR interval differences; HF in ms^2^, absolute power of the high-frequency (HF) band; HF in n.u., relative power of HF band; SD1, Poincaré plot standard deviation perpendicular to the line of identity; PNS, Index, parasympathetic nervous system tone index. * = statistically significant at *p* < 0.05.

**Table 4:** Baseline values of cognitive tests and DASS-21—score of subscale ‘depression’ in mNCD population.

	Unit	mNCD (*n* = 29)
PEBL-DSB	score	4.08 ± 1.61
PEBL-DSB	maximum span	3 (1)
PEBL-TMT-B	seconds	139.82 ± 81.01
HOTAP	points/min	5.08 ± 2.11
Depression	score	3 (2.25)

Data are reported as mean ± standard deviation for data fulfilling all the assumptions that would subsequently justify parametric statistical analyses data and as median (IQR) for data not fulfilling all of these assumptions.

### 3.2 Primary outcome: diagnostic accuracy of vm-HRV

The results on AUCs, sensitivities, and specificities for HRV parameters are presented in Table 5. The greatest AUC calculated was 0.68 (with 95% CI: 0.56, 0.81) for the relative power of HF. The remaining HRV parameters had AUCs between 0.61 and 0.53. Six HRV parameters showed sensitivity above 62%, with specificity ranging widely from 26 to 76%.

**Table 5:** Diagnostic accuracy of vm-HRV.

HRV parameter	AUC (CI)	Sensitivity	Specificity
HF in n.u.	0.68 (0.56, 0.81)	69%	67%
pnn50	0.61 (0.47, 0.74)	62%	42%
PNS-Index	0.56 (0.42, 0.70)	69%	51%
SD1	0.53 (0.85, 0.69)	68%	49%
mRR	0.53 (0.40, 0.67)	86%	26%
RMSSD	0.53 (0.38, 0.69)	68%	49%
HF in ms2	0.53 (0.37, 0.68)	42%	76%

Area under the curve (AUC) with confidence interval (CI = 95%), sensitivity (%), and specificity (%).

### 3.3 Secondary outcome: vm-HRV and executive functions and depression

Multiple linear regression showed a significant correlation between vm-HRV and executive functioning with regards to planning abilities and mRR (R^2^ = 0.38, *p* < 0.05) as well as relative power of HF (R^2^ = 0.42, *p* < 0.05; Table 6), but not cognitive flexibility (Table 7) or working memory (Table 8).

**Table 6:** Multiple linear regression models with HOTAP as the constant outcome variable, vm-HRV parameters as predictor variables, and physical activity (MET-min/week) and education as constant covariates.

	R^2^	B	SE B	β	*p* value	Covariates
Executive function: HOTAP						
mRR	0.38	0.01	0.00	0.36	0.038*	PA, education
RMSSD	0.39	−0.04	0.03	−0.25	0.18	PA, education
pNN50	0.45	−0.04	0.07	−0.09	0.59	PA, education
HF	0.45	0.00	0.00	−0.03	0.85	PA, education
HF in n.u.	0.42	−0.04	0.01	−0.42	0.015*	PA, education
SD1	0.39	−0.05	0.04	−0.25	0.18	PA, education
PNS	0.34	0.31	0.29	0.19	0.30	PA, education

R^2^, coefficient of determination; B, regression coefficient; SE, standard error of B; β, standardized B; PA, physical activity. * = statistically significant at *p* < 0.05.

**Table 7:** Multiple linear regression models with trail-making task part B as the constant outcome variable, vm-HRV parameters as predictor variables.

	R^2^	B	SE B	β	*p* value	Covariates
Executive function: trail-making task part B						
mRR	0.29	−0.17	0.10	−0.30	0.11	Sex, PA
RMSSD	0.35	1.23	1.08	0.21	0.27	Sex, PA
pNN50	0.37	−1.25	3.06	−0.08	0.69	Sex, PA
HF	0.36	−0.01	0.12	−0.02	0.92	Sex, PA
HF in n.u.	0.23	0.58	0.66	0.18	0.39	Sex, PA
SD1	0.35	1.74	1.52	0.21	0.26	Sex, PA
PNS	0.31	−3.90	11.14	−0.06	0.73	Sex, PA

R^2^, coefficient of determination; B, regression coefficient; SE, standard error of B; β, standardized B; PA, physical activity.

**Table 8:** Multiple linear regression models with digit span backward as the constant outcome variable, vm-HRV parameters as predictor variables, and physical activity (MET-min/week) and education as constant covariates.

	R^2^	B	SE B	*β*	*p* value	Covariates
Executive function: digit span backward (total point score)						
mRR	0.13	0.00	0.00	0.09	0.67	PA, education
RMSSD	0.22	−0.04	0.02	−0.33	0.13	PA, education
pNN50	0.14	0.00	0.07	−0.01	0.98	PA, education
HF	0.16	0.00	0.00	−0.12	0.60	PA, education
HF in n.u.	0.12	0.00	0.01	−0.03	0.90	PA, education
SD1	0.22	−0.05	0.03	−0.33	0.13	PA, education
PNS	0.13	−0.10	0.26	−0.08	0.71	PA, education
Executive function: digit span backward (max. span)						
mRR	0.13	0.00	0.00	0.004	0.98	PA, education
RMSSD	0.09	−0.01	0.01	−0.04	0.54	PA, education
pNN50	0.07	0.00	0.04	−0.03	0.91	PA, education
HF	0.08	0.00	0.00	−0.11	0.64	PA, education
HF in n.u.	0.15	0.01	0.01	0.17	0.41	PA, education
SD1	0.09	−0.01	0.02	−0.14	0.54	PA, education
PNS	0.07	−0.01	0.14	−0.02	0.93	PA, education

R^2^, coefficient of determination; B, regression coefficient; SE, standard error of B; β, standardized B; PA, physical activity.

DASS-21 - score of subscale ‘depression’ was also not related to vm-HRV parameters (Table 9) in patients with mNCD.

**Table 9:** Multiple linear regression models with DASS-21—score of subscale ‘depression’ as the constant outcome variable, vm-HRV parameters as predictor variables.

	R^2^	B	SE B	*β*	*p* value	Covariates
Depression						
mRR	0.07	0.00	0.00	0.07	0.71	Sex, education
RMSSD	0.14	−0.02	0.05	−0.10	0.63	Sex, education
pNN50	0.24	−0.27	0.14	−0.39	0.07	Sex, PA
HF	0.20	−0.01	0.01	−0.32	0.14	Sex, PA
HF in n.u.	0.15	−0.04	0.03	−0.32	0.14	Sex, education
SD1	0.14	−0.04	0.07	−0.10	0.63	Sex, education
PNS	0.17	−0.62	0.55	−0.23	0.27	Sex, education

Physical activity (MET-min/week) and gender were covariates in pnn50 and absolute HF. Education and gender were covariates in all other vm-HRV parameters. R^2^, coefficient of determination; B, regression coefficient; SE, standard error of B; β, standardized B; PA, physical activity.

## 4 Discussion

The aim of this study was to evaluate the diagnostic accuracy of vm-HRV as a screening tool for mNCD and investigate its relationship with executive functioning and depression in this population. The relative power of HF had the highest AUC, but all evaluated vm-HRV parameters showed poor diagnostic accuracy. Mixed findings were observed for the relationship between vm-HRV and executive functioning, while no relation was found between vm-HRV and depressive symptoms.

### 4.1 Diagnostic accuracy of vm-HRV

This study was the first to investigate the diagnostic accuracy of vm-HRV parameters as a screening tool for mNCD, precluding direct comparisons with the literature. However, previous literature has shown that vm-HRV parameters are sensitive to mNCD, with lower values in individuals with mNCD than in healthy controls (da Silva et al., 2018; Cheng et al., 2022). This is consistent with the predictions of the “neurovisceral integration” model, which suggests that vm-HRV can indicate the functional integrity of the CAN implicated in cognitive and emotional responses to the environment (Thayer, 2009). However, it is inconsistent with our findings, as we observed mixed results, including increased (rather than the expected decreased) resting HF-HRV in individuals with mNCD. This finding may be explained by the study of Lin et al. suggesting the presence of compensatory mechanisms in cognitively impaired patients (Lin et al., 2017a). They found that worse cognitive performance and more severe neurodegeneration in individuals with mNCD were associated with greater HF-HRV during rest, and suggested a framework of a non-linear relationship between parasympathetic nervous system (PNS) activity (i.e., vm-HRV) and neurodegeneration (Lin et al., 2017b).

In individuals with mNCD, the increased level of PNS activity compared to HOA may be caused by hyperactivity of brain regions [i.e., anterior cingulate cortex (ACC)] as a result of insufficient neural efficiency of frontal regions or a compensatory mechanism for neural loss in frontal regions (Li, Cao et al., 2014; Lin et al., 2017a). Yetkin et al. observed that both patients with MCI and patients with AD displayed more activation than healthy controls in the frontal and temporal lobes (Yetkin et al., 2006). Additionally, Yoon et al. showed hyperactivation of the prefrontal cortex (PFC) in MCI during a cognitive task suggesting the presence of neural compensatory mechanisms (Yoon et al., 2019). The PFC, the ACC, the amygdala, the insula, the hypothalamus, and the brainstem are all part of CAN, a component of the body’s internal regulation system. This integrated network is mediated by preganglionic sympathetic and parasympathetic (vagal) neurons (Benarroch, 1993; Thayer et al., 2012; Shouman and Benarroch, 2021) which innervate the heart and regulate HRV (Shaffer et al., 2014). The brain areas in CAN are shown to be impaired in MCI (Van Dam et al., 2013; Xu et al., 2020) rendering the utilization of compensatory mechanisms which are then reflected in changes in HRV. This provides a possible explanation for the increased HF-HRV in mNCD individuals at an early stage of the disease.

The model proposed by Lin et al. (2017b) also aligns with the evidence on the sensitivity of vm-HRV for mNCD, based on our preliminary comparison of this study and others involving patients with neurodegenerative disorders (Lin et al., 2017a; da Silva et al., 2018; Nicolini et al., 2022; Grässler et al., 2023). In particular, in our comparison the decline in global cognitive function was associated with relative changes in vm-HRV (ΔHF-HRV in n.u.) corresponding to neurodegeneration (Ye et al., 2017; Radanovic et al., 2019) and PNS activity, respectively. Among the reviewed studies, three included patients diagnosed with mNCD (Lin et al., 2017b; Nicolini et al., 2022; Grässler et al., 2023), five involved patients with Alzheimer’s Disease (da Silva et al., 2018), and two focused on patients with Lewy Body Dementia (da Silva et al., 2018). All studies, except ours, used multi-lead ECG to measure vm-HRV (Lin et al., 2017a, da Silva et al., 2018, Nicolini et al., 2022, Grässler et al., 2023). While these findings show some alignment with the framework proposed by Lin et al. (2017b), further research is needed to confirm or reject this preliminary framework by including a broad range of neurocognitive disorders, HRV measurements, and neuroimaging approaches.

### 4.2 Vm-HRV and executive functions and depression

This study did not find consistent correlations between vm-HRV and executive functioning in the mNCD group, which contrasts with previous literature (Forte et al., 2019; Liu et al., 2022). Our findings can be explained in the light of the previously discussed framework by Lin et al. (2017a). The relationship between PNS activity and neurodegeneration is suggested to be non-linear, which can be compared to the relationship between vm-HRV and executive function performance, as deficits in cognitive performance are known to correlate with neurodegeneration (Ye et al., 2017). Therefore, future investigations should employ new methodologies to capture the interaction between vm-HRV and cognitive performance, taking into account the potential non-linear relationship, and elucidate whether the potential relationship between executive functions and vm-HRV is dependent on neurocognitive subdomains of executive function and the associated brain structures and networks involved.

This study’s findings on the missing relationship between vm-HRV and depression also contradicts previous literature (Kemp et al., 2010; Ha et al., 2015). A possible explanation for this discrepancy is that 20% of the patients in our study scored the lowest possible score on the DASS-21 subscale for depression (score = 0). Additionally, the study revealed that 76% of the patients scored 4 or lower on the DASS-21 depression subscale, indicating an absence of clinically relevant depressive symptoms in these individuals (Lovibond, 1995). Terwee et al. suggested that the floor (bottom) effect may be present if more than 15% of participants achieve the lowest possible score (Terwee et al., 2007). Therefore, it can be concluded that a bottom effect is present in the data for the DASS-21 subscale ‘depression’, which may explain the lack of a correlation between vm-HRV and depression. This bottom effect may be due to potential selection bias in the recruitment process (see section ‘Strengths and Limitations of the Study and Future Research’).

### 4.3 Generalizability of the findings

Compared to previous studies investigating vm-HRV parameters in relation to cognitive performance (Lin et al., 2017a, Grässler et al., 2021b, Nicolini et al., 2022), our study population had similar descriptive statistics for age (only for either HOA or mNCD), sex distribution (only HOA), BMI (both groups), and education [only study of Lin et al. (2017b)], which makes our study population comparable to previous research.

However, it is important to note that the generalizability of our findings may be limited by certain characteristics of our populations. Both the mNCD and healthy group exhibited a moderate level of physical activity (Forde, 2018), with higher IPAQ scores compared to the mNCD and healthy participants in a reference study (Makarewicz et al., 2021). It is well-known that higher levels of physical activity are associated with higher HRV indices in the older adults population (Buchheit et al., 2004), which indicates a highly active population and may limit the generalizability of our findings. In comparison to previous studies, our study population had more years of education, except compared to the Lin et al. (2017a). Higher levels of education in early life are recognized as protective factors against neurocognitive disorders, potentially associated with enhanced cognitive performance and cognitive reserve (Stern, 2012; Stern et al., 2020; Zhang et al., 2022). In addition, it remains uncertain whether years of education impact vm-HRV in older adults (Chuang et al., 2018). However, as our study was conducted on a well-educated population it may limit the generalizability of our findings in individuals with lower levels of education. Additionally, women were underrepresented in the studied population. Even though the vm-HRV results should not be affected by this factor as sex differences in vm-HRV disappear after the age of 55 (Voss et al., 2015), the prevalence of non-amnestic mNCD is higher in women, which may limit the generalizability of our findings (Au et al., 2017). Moreover, our study population was mainly comprised of well-educated and active men with mNCD. This could again be due to a selection bias in participant recruitment, as the study only included individuals referred to us by our clinical collaborators. Additionally, the 12-week training intervention design of the study may have introduced a bias toward against active individuals, due to the heightened barriers to enrolling in a 12-week intervention study as opposed to a typical cross-sectional study utilized for evaluating the diagnostic accuracy of a screening tool.

The significant age difference between the analyzed populations should also be taken into consideration. Previous research suggests that vm-HRV decreases with age until the sixth decade of life and then stabilizes (De Meersman and Stein, 2007; Voss et al., 2012). Furthermore, Stein et al. (2009) demonstrated the age-related decline of parasympathetic control of the heart levels at age 70 (Stein et al., 2009). Therefore, the significant between groups difference in terms of age should not have a relevant impact on our result and does not limit the generalizability of our findings. On the other hand, our study provides a highly representative sample of the distribution of mNCD etiologies. Alzheimer’s disease was the most common etiology among participants with mNCD, accounting for 72% of cases. The mild vascular neurocognitive disorder is the second most common etiology (17%), followed by mild frontotemporal neurocognitive disorder etiology (7%) and Lewy Body dementia etiology (3%), which is consistent with the expected distribution of etiologies in populations with mNCD (American Psychiatric Association, 2013).

Taking all these factors into account, the generalizability of our findings applies to a moderately active and well-educated population, with the inclusion of various etiologies of mNCD. However, the generalizability of our findings may be limited to less active, less educated individuals, and women. Therefore, it is necessary to verify our findings by testing in more representative populations of individuals with mNCD.

### 4.4 Implication for research

Our key findings demonstrate that vm-HRV alone does not seem to possess sufficient diagnostic accuracy for mNCD. In this regard, a multimodal approach that considers a variety of functional domains impaired in a certain disorder may present an interesting avenue for improving this diagnostic accuracy. For instance, cognitive dysfunction has a negative impact on other functional domains, such as gait parameters, dual-task performance, or chemosensory functioning (i.e., olfactory function). More specifically, balance, gait speed (Kuan et al., 2021), and gait variability (Pieruccini-Faria et al., 2021) are impaired in patients with mNCD and may be linked to discrete neural changes in the brain (Wilson et al., 2019) that may support distinguishing between different mNCD etiologies. Similarly, MCI is associated with olfactory impairments which were more severe in patients with amnestic MCI than patients with non-amnestic MCI (Zhou et al., 2024). In addition, innovative digital cognitive screening modalities, such as smart devices (e.g., smartphones, smartwatches, or smart-home devices), serious games, or virtual reality might improve diagnostic accuracy of screening of cognitive impairment and reduce feelings of test anxiety (Magno et al., 2024). These could also implement dual-task walking procedures, which have been shown as a useful predictor of cognitive impairment and performed better than single-task gait measurements alone (Ramírez and Gutiérrez, 2021). More and more research tends to focus on non-invasive measurements of functional brain changes. Electroencephalography and functional near-infrared spectroscopy measurements have been shown useful and therefore suggested as a screening tool to distinguish between HOA and individuals with MCI and Alzheimer’s disease (Yeung and Chan, 2020; Jiao et al., 2023).

While all these modalities might be relevant, they typically only cover specific aspects of mNCD. Therefore, it has recently been proposed that combining different noninvasive measurement modalities might improve diagnostic accuracy for early detection of mNCD/MCI (Grässler et al. 2021a), with some preliminary evidence supporting this assumption (e.g., combining bioimpedance analysis with a standard cognitive screening instrument improved diagnostic accuracy for detecting MCI compared to cognitive screening alone; Jun et al., 2024). Thus, combining these (and other) modalities with vm-HRV in future research renders a promising implication for screening for mNCD and should be considered as a starting point for developing innovative screening tools.

The findings of this study may have implications for differentiating mNCD from other diseases. It has been observed that vm-HRV is decreased in disorders such as depression (Kemp et al., 2010) and dementia (da Silva et al., 2018; Cheng et al., 2022), but might be increased in mNCD. This could potentially allow future research to differentiate mNCD from other neurocognitive and psychological disorders. However, it is crucial to verify the findings and proposed framework of Lin et al. (2017b) by conducting studies that carefully examine this aspect. Therefore, we recommend conducting longitudinal studies to observe the change in HRV over time in a wide range of neurocognitive disorders, as well as combining this with neuroimaging approaches.

### 4.5 Strengths and limitations of the study and future research

A key strength of this study is the selective inclusion of participants who have a clinical diagnosis of mNCD according to ICD-XI (World Health Organization, 2018) or DSM-5 (American Psychiatric Association, 2013) with a biomarker-supported characterization of the etiology of mNCD rendering our population a gold standard for mNCD research. Furthermore, the study encompasses data from individuals with diverse mNCD etiologies which increases the generalizability of our findings.

Although our study has provided valuable strengths, it is important to acknowledge certain limitations. Firstly, the intervention study design may have introduced selection bias in participant recruitment, particularly in less active individuals and those with mNCD. This may impact the generalizability of our findings. Secondly, as this is a retrospective study, we only included individuals who had already been diagnosed with mNCD at the time of HRV measurement which may impact the robustness of our findings and only allow cautious preliminary conclusions. To mitigate this limitation, it would be beneficial to measure a healthy, but at risk for cognitive impairment, population and then follow up and investigate whether HRV can discriminate between individuals who remain healthy and those who subsequently develop mNCD. Additionally, this study is limited by being a secondary analysis of three existing studies, and neither their design nor their sample size was tailored to the research objective of this study, which may also limit the robustness of the findings and only allow cautious preliminary conclusions. Furthermore, our analysis did not differentiate between the types of medication taken by participants, since such data was only available for the second cohort of participants with mNCD. The influence of medication should be analyzed in the future to correct for potential confounding effects of medication on the observed HRV patterns instead of only the (lack of) intake of cardioactive medications. To address these limitations in future research and allow more robust and nuanced conclusions, a prospective study with a carefully calculated sample size and a thorough examination of medication effects on HRV would provide a more comprehensive and robust understanding of the relationships under investigation. Finally, this study was, to the best of our knowledge, the first to investigate vm-HRV in individuals with a clinical diagnosis of mNCD with a biomarker-supported characterization of four distinct etiologies of mNCD. Unfortunately, the available data did not allow subgroup analyses to elucidate whether these distinct etiologies affect the brain-heart axis differentially due to the low number of patients with etiologies other than Alzheimer’s disease, highlighting the need for further research in this area to allow more nuanced conclusions.

## 5 Conclusion

In conclusion, it appears that vm-HRV parameters alone are insufficient to reliably distinguish between HOA and older adults with mNCD. Furthermore, the relationship between vagally-mediated heart rate variability and executive functioning remains unclear and requires further investigation. Our findings warrant prospective studies that encompass a broad range of neurocognitive disorders, HRV measurements, neuroimaging approaches, and a multifactorial approach to further investigate the potential of vm-HRV as a screening tool for mNCD. These steps have the potential to improve the early detection of mNCD in the future.

## Data Availability

Publicly available datasets were analyzed in this study. This data can be found at: https://doi.org/10.5281/zenodo.7824568, https://doi.org/10.5281/zenodo.7428378, and https://doi.org/10.5281/zenodo.10695988.
