# Supplementary material for: Diagnostic accuracy of heart rate variability as a screening tool for mild neurocognitive disorder
Source: Front Aging Neurosci. 2024 Dec 17;16:1498687. doi: 10.3389/fnagi.2024.1498687 (PMC11685156; doi:10.3389/fnagi.2024.1498687)
Supplement: Supplementary file 1 [file Data_Sheet_1.pdf]

Supplementary Material to Publication:

Diagnostic Accuracy of Heart Rate Variability as a Screening Tool for Mild Neurocognitive Disorder

Julia Czopek-Rowinska[1], Eling D. de Bruin[1-3], Patrick Manser[1*]

[1]Motor Control and Learning Lab, Institute of Human Movement Sciences and Sport, Department of Health Sciences and Technology, ETH Zurich, Zurich, Switzerland

[2]Department of Health, OST - Eastern Swiss University of Applied Sciences, St. Gallen, Switzerland;

[3]Division of Physiotherapy, Department of Neurobiology, Care Sciences and Society, Karolinska Institutet, Stockholm, Sweden

*** Correspondence:**
Patrick Manser
patrick.manser@hest.ethz.ch / patrick.manser@ki.se

Supplementary File 1 - Standards for Reporting of Diagnostic Accuracy Studies Checklist [1, 2]

Section & Topic	No	Item	Reported in paragraph
**TITLE OR ABSTRACT**			
	**1**	Identification as a study of diagnostic accuracy using at least one measure of accuracy (such as sensitivity, specificity, predictive values, or AUC)	Abstract
**ABSTRACT**			
	**2**	Structured summary of study design, methods, results, and conclusions (for specific guidance, see STARD for Abstracts)	Abstract
**INTRODUCTION**			
	**3**	Scientific and clinical background, including the intended use and clinical role of the index test	Introduction
	**4**	Study objectives and hypotheses	Introduction
**METHODS**			
Study design	**5**	Whether data collection was planned before the index test and reference standard were performed (prospective study) or after (retrospective study)	Study design and setting
Participants	**6**	Eligibility criteria	Eligibility criteria
	**7**	On what basis potentially eligible participants were identified (such as symptoms, results from previous tests, inclusion in registry)	Study design and setting
	**8**	Where and when potentially eligible participants were identified (setting, location and dates)	Study design and setting
	**9**	Whether participants formed a consecutive, random or convenience series	Study design and setting
Test methods	**10a**	Index test, in sufficient detail to allow replication	Outcomes
	**10b**	Reference standard, in sufficient detail to allow replication	N/A
	**11**	Rationale for choosing the reference standard (if alternatives exist)	N/A
	**12a**	Definition of and rationale for test positivity cut-offs or result categories of the index test, distinguishing pre-specified from exploratory	Statistics
	**12b**	Definition of and rationale for test positivity cut-offs or result categories of the reference standard, distinguishing pre-specified from exploratory	N/A
	**13a**	Whether clinical information and reference standard results were available to the performers/readers of the index test	N/A
	**13b**	Whether clinical information and index test results were available to the assessors of the reference standard	N/A
Analysis	**14**	Methods for estimating or comparing measures of diagnostic accuracy	Statistics
	**15**	How indeterminate index test or reference standard results were handled	Statistics
	**16**	How missing data on the index test and reference standard were handled	Statistics
	**17**	Any analyses of variability in diagnostic accuracy, distinguishing pre-specified from exploratory	N/A
	**18**	Intended sample size and how it was determined	Eligibility criteria
**RESULTS**			
Participants	**19**	Flow of participants, using a diagram	N/A
	**20**	Baseline demographic and clinical characteristics of participants	Participants
	**21a**	Distribution of severity of disease in those with the target condition	Baseline data
	**21b**	Distribution of alternative diagnoses in those without the target condition	N/A
	**22**	Time interval and any clinical interventions between index test and reference standard	N/A
Test results	**23**	Cross tabulation of the index test results (or their distribution) by the results of the reference standard	N/A
	**24**	Estimates of diagnostic accuracy and their precision (such as 95% confidence intervals)	Primary Outcome
	**25**	Any adverse events from performing the index test or the reference standard	N/A
**DISCUSSION**			
	**26**	Study limitations, including sources of potential bias, statistical uncertainty, and generalisability	Strengths and Limitations
	**27**	Implications for practice, including the intended use and clinical role of the index test	Implications for research
**OTHER INFORMATION**			
	**28**	Registration number and name of registry	N/A

	**29**	Where the full study protocol can be accessed	N/A
	**30**	Sources of funding and other support; role of funders	N/A

References

1. Bossuyt PM, Reitsma JB, Bruns DE, Gatsonis CA, Glasziou PP, Irwig L, Lijmer JG, Moher D, Rennie D, de Vet HCW *et al*: **STARD 2015: an updated list of essential items for reporting diagnostic accuracy studies**. *BMJ : British Medical Journal* 2015, **351**:h5527.
2. Cohen JF, Korevaar DA, Altman DG, Bruns DE, Gatsonis CA, Hooft L, Irwig L, Levine D, Reitsma JB, Vet HCWd *et al*: **STARD 2015 guidelines for reporting diagnostic accuracy studies: explanation and elaboration**. *BMJ Open* 2016, **6**(11):e012799.
